# Correction: Synthesis, and single crystal structure of fully-substituted polynitrobenzene derivatives for high-energy materials

**DOI:** 10.1039/c8ra90009d

**Published:** 2018-02-06

**Authors:** Wei Yang, Huanchang Lu, Longyu Liao, Guijuan Fan, Qing Ma, Jinglun Huang

**Affiliations:** Institute of Chemical Materials, China Academy of Engineering Physics Mianshan Road 64 Mianyang China fanguijuan@caep.cn; Department of Chemistry and Chemical Biology, Harvard University Cambridge Massachusetts 02138 USA

## Abstract

Correction for ‘Synthesis, and single crystal structure of fully-substituted polynitrobenzene derivatives for high-energy materials’ by Wei Yang *et al.*, *RSC Adv.*, 2018, **8**, 2203–2208.

The literature citation to molecule **1** in Scheme 1 was in error and should have been to ref. 8; the corrected Scheme 1 is attached.

**Scheme 1:**
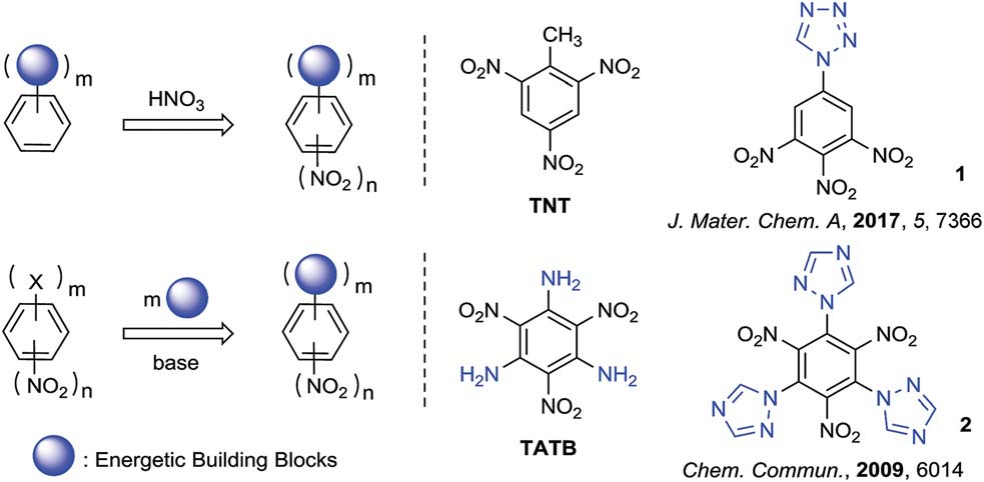
Synthetic strategy of polynitrobenzene derivatives for energetic materials.

The Royal Society of Chemistry apologises for these errors and any consequent inconvenience to authors and readers.

## Supplementary Material

